# A Novel Method for Classifying Driver Mental Workload Under Naturalistic Conditions With Information From Near-Infrared Spectroscopy

**DOI:** 10.3389/fnhum.2018.00431

**Published:** 2018-10-26

**Authors:** Anh Son Le, Hirofumi Aoki, Fumihiko Murase, Kenji Ishida

**Affiliations:** ^1^Human Factors and Aging Laboratory, Institutes of Innovation for Future Society, Nagoya University, Nagoya, Japan; ^2^Department of Power Engineering, Faculty of Engineering, Vietnam National University of Agriculture, Hanoi, Vietnam; ^3^Denso Corporation, Nisshin, Japan

**Keywords:** near-infrared spectroscopy, cognitive distraction, classification, driver attention, mental workload, artificial intelligence

## Abstract

Driver cognitive distraction is a critical factor in road safety, and its evaluation, especially under real conditions, presents challenges to researchers and engineers. In this study, we considered mental workload from a secondary task as a potential source of cognitive distraction and aimed to estimate the increased cognitive load on the driver with a four-channel near-infrared spectroscopy (NIRS) device by introducing a machine-learning method for hemodynamic data. To produce added cognitive workload in a driver beyond just driving, two levels of an auditory presentation n-back task were used. A total of 60 experimental data sets from the NIRS device during two driving tasks were obtained and analyzed by machine-learning algorithms. We used two techniques to prevent overfitting of the classification models: (1) *k*-fold cross-validation and principal-component analysis, and (2) retaining 25% of the data (testing data) for testing of the model after classification. Six types of classifier were trained and tested: decision tree, discriminant analysis, logistic regression, the support vector machine, the nearest neighbor classifier, and the ensemble classifier. Cognitive workload levels were well classified from the NIRS data in the cases of subject-dependent classification (the accuracy of classification increased from 81.30 to 95.40%, and the accuracy of prediction of the testing data was 82.18 to 96.08%), subject 26 independent classification (the accuracy of classification increased from 84.90 to 89.50%, and the accuracy of prediction of the testing data increased from 84.08 to 89.91%), and channel-independent classification (classification 82.90%, prediction 82.74%). NIRS data in conjunction with an artificial intelligence method can therefore be used to classify mental workload as a source of potential cognitive distraction in real time under naturalistic conditions; this information may be utilized in driver assistance systems to prevent road accidents.

## Introduction

Driver distraction is a major cause of traffic accidents (NHTSA, 2015). An analysis by the US Highway Traffic Safety Administration (NHTSA) showed that driver distraction can be categorized into three types: visual distraction, manual distraction, and cognitive distraction (NHTSA, 2012). Among these, cognitive distraction is the most difficult type to address, because it occurs within the driver's brain (Rizzo and Hurtig, 1987; Engström et al., 2005; Angell et al., 2006). Cognitive distraction is defined as the mental workload associated with a task that involves thinking about something other than driving. The detection of cognitive distraction imposed by a secondary task while driving might play an important role in creating a new driver-assistance system to reduce the incidence of traffic accidents.

Dong et al. (2011) categorized techniques for measuring mental workload while driving into five groups: (1) subjective metrics, (2) biological metrics, (3) physical metrics, (4) performance metrics, and (5) combinations of these metrics. Because the central goal of our research was to identify and improve a metric that might permit the detection of mental workload in real time and which could operate under real conditions in the presence of, for example, vibration from the vehicle, we examined only physical metrics in the present study.

One potential physical metric involves the use of eye-movement information. Many researchers have previously attempted to identify a relationship between mental workload and various items of information on the eye, such as blink (Tsai et al., 2007; Benedetto et al., 2011), pupil diameter (Backs and Walratht, 1992; Klingner et al., 2008; Schwalm et al., 2008; Klingner, 2010), saccades (Tsai et al., 2007; Pierce, 2009; Tokuda et al., 2009), gaze concentration (Wang et al., 2014), or eye fixation (Klingner, 2010). Each of these methods has its advantages and disadvantages. For example, pupil diameter has a strong relationship to the level of cognitive load but it is also highly sensitive to the frequent changes in light that occur while driving (Palinko and Kun, 2012). Another potential method is to use the involuntary eye movements based on the vestibulo-ocular reflex model that are simulated by head movements or by vibrations from the moving vehicle. In this method, differences between predicted and actual eye simulation are assessed as a measure of mental workload (Obinata et al., 2008, 2009, 2010; Aoki et al., 2015; Anh Son et al., 2016, 2017a,b,c,d,e, 2018; Le and Aoki, 2018; Son and Hirofumi, 2018; Son et al., 2018) However, the use of eye information to measure mental workload still has some limitations, such as oversensitivity to light, vibration, noise, and visual information.

In terms of a physical metric, monitoring of brain activity by electroencephalography or the use of the heart rate as an indicator of mental workload have been confirmed to be effective (Meshkati, 1988; Lee and Park, 1990; Jorna, 1992; Porges and Byrne, 1992; Veltman and Gaillard, 1996; Ryu and Myung, 2005; Henelius et al., 2009; Mehler et al., 2012; Cinaz et al., 2013; Angell and Perez, 2015). However, these techniques require physical attachment of the monitoring equipment and are highly sensitive to the driver age, body position, and muscle activity.

One method with a high potential for application is the use of information from near-infrared spectroscopy (NIRS) to classify mental workload (Kopton and Kenning, 2014). NIRS has been used in various fields; for example, in agriculture to check the quality of crops and in medicine to assess oxygenation and microvascular function. In terms of classifying mental workload, a relationship between mental workload and activity of the central nervous system has been confirmed by McBride and Schmorrow (2005). Since their work, other researchers have attempted to classify levels of mental workload by applying artificial-intelligence analyses (Tsunashima and Yanagisawa, 2009; Herff et al., 2014; Ichikawa et al., 2014; Aghajani et al., 2017). All of these researchers showed that NIRS has considerable potential in quantifying mental workload while driving, especially in naturalistic cases (Kopton and Kenning, 2014; Liu et al., 2016).

Furthermore, Toshinori Kato and his group have done various investigations on NIRS data, especially how to filter the signals and map it (Kohri et al., 2002; Yoshino et al., 2013, 2015; Orino et al., 2015). In actual driving, his group pointed out that there was a relationship of the brain activity with the vehicle speed. Their research also confirmed that fNIRS data is one of a good solution for monitoring the driver status while driving especially in actual condition. Further, Liu et al. (2017) confirmed that the cognitive workload has a relationship with the hemodynamic activity level (Liu et al., 2017). His team also mentioned that the effective association can be weak in case of driving with subtasks. However, none of them investigated in applying machine learning with raw data to detect mental workload by secondary task while driving.

The central goal of our study was to examine whether or not it is possible to classify driver mental workload by using supervised learning with NIRS data obtained in a real vehicle. In this report, we initially point out the importance of detecting cognitive load while driving. We then review and summarize methods for evaluating cognitive workload that have been reported in the literature. We also discuss the differences in mental workload between doing one task and driving with secondary task. We then review the use of NIRS information to classify cognitive load, and we describe our experimental design and methods for analyzing NIRS data. The result of the classification are reported and, finally, we discuss our conclusions and any challenges that remain.

## Materials and methods

### Experimental design

**Figure 1:**
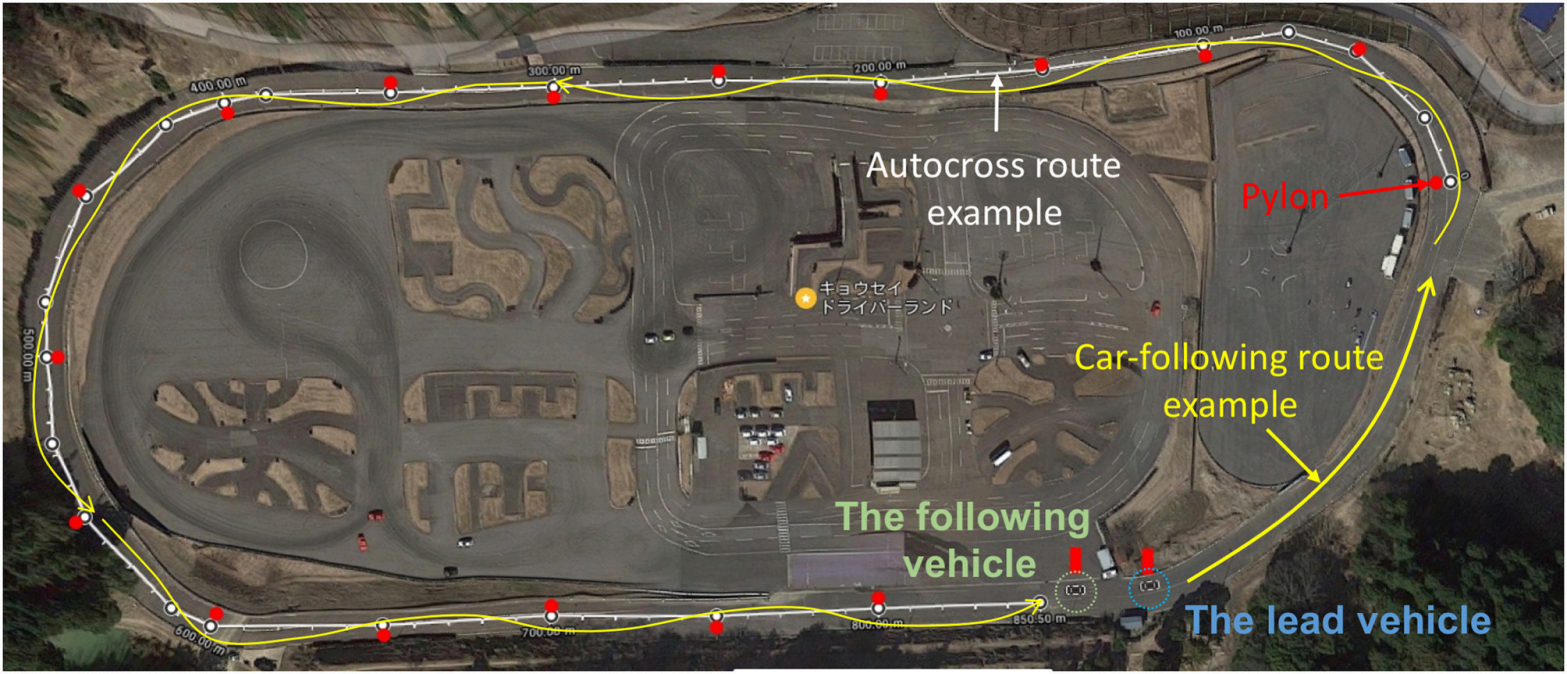
Test course.

One female and four male subjects, who each held a driver's license (mean age: 38 ± 10; two professional drivers, and three newly qualified drivers), were recruited for this test. A total of 60 experiments were performed involving navigating a defined course alone (autocross) or following another vehicle (car-following) on a test course (Figure 1). The experiments were approved by the Ethical Review Board of Nagoya University's Institute of Innovation for Future Society. All subjects were provided with explanations regarding the experimental procedure, and all gave their written informed consent.

**Figure 2:**
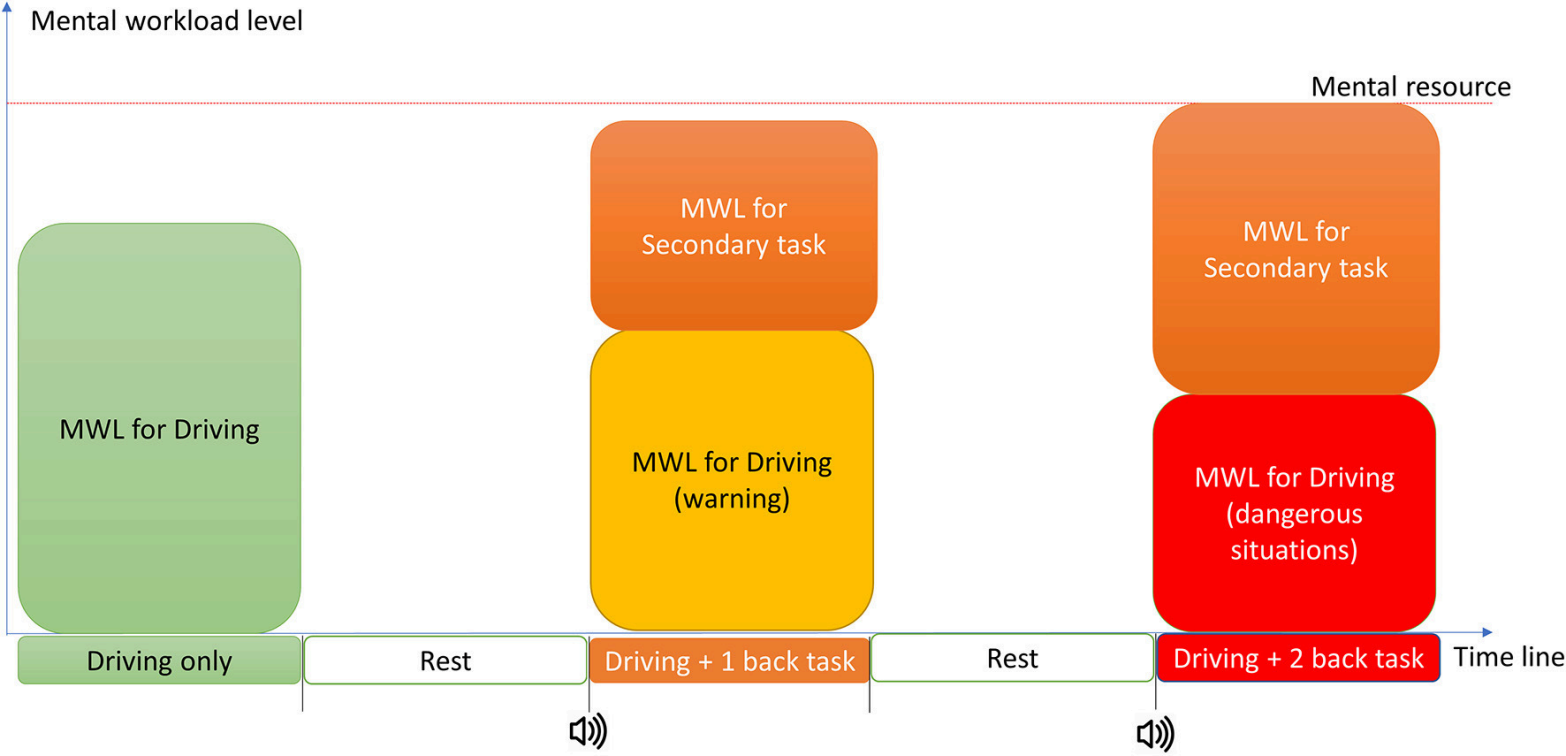
Experiment procedure and hypothetical image of MWL.

The experiment procedure is shown in Figure 2. The subjects performed a series of tests involving a driving task (autocross or car following) during which drivers were asked to drive around 40 km/h. For portions of these drives, additional mental workload was introduced by asking subjects to engage in two levels of an n-back auditory digit recall task in which a number was verbally presented to the subject every 2 s. In the 1-back test, the subject was asked to press the “Yes” button when the number heard was the same as the previous one or the “No” button when it was different. In the 2-back task, the subject similarly had to remember the number preceding the previous one. The “Yes” and “No” buttons were installed on the driving wheel so they could be easily pressed.

**Figure 3:**
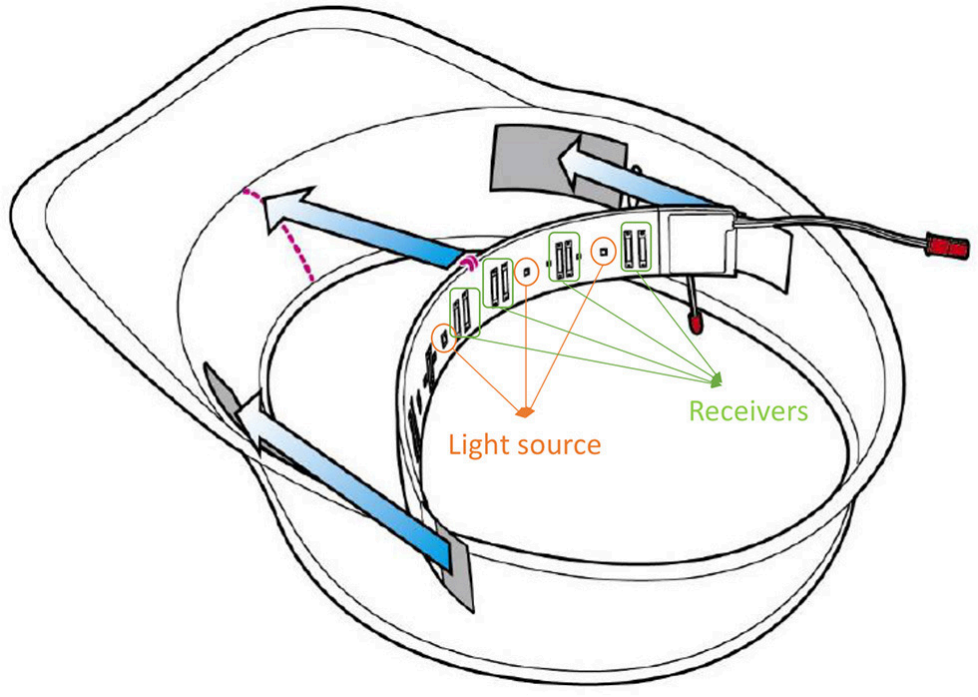
ASTEM's NIRS device.

As our main aim is to create an algorithm for an advanced driver-assistance system to help prevent traffic accidents by identifying driver cognitive distraction, the classification needed to be reliable, quick, and easy. To achieve this, we used a commercial four-channel NIRS system (Astem Corp., Fukuoka, Japan) which was placed on the forehead of the subject, where the signals from the four channels are almost the same (Figure 3). This device can measure blood oxyhemoglobin (oxy-Hb) and deoxyhemoglobin (deoxy-Hb) levels at wavelengths of 770 nm (probe distance 35 mm) and 830 nm (probe distance is 40 mm), and oxygen saturation at 35 mm.

Furthermore, to keep the class-balance data set for machine learning, the subjects were asked to drive around the course for each task (driving task only, driving plus 1-back task, and driving plus 2-back task), and repeat it twice (average time for each trial was 1 min 54 ± 16 s depending upon the speed actually driven). Therefore, the sample data input to machine learning step was class-balance.

### Data processing

**Figure 4:**
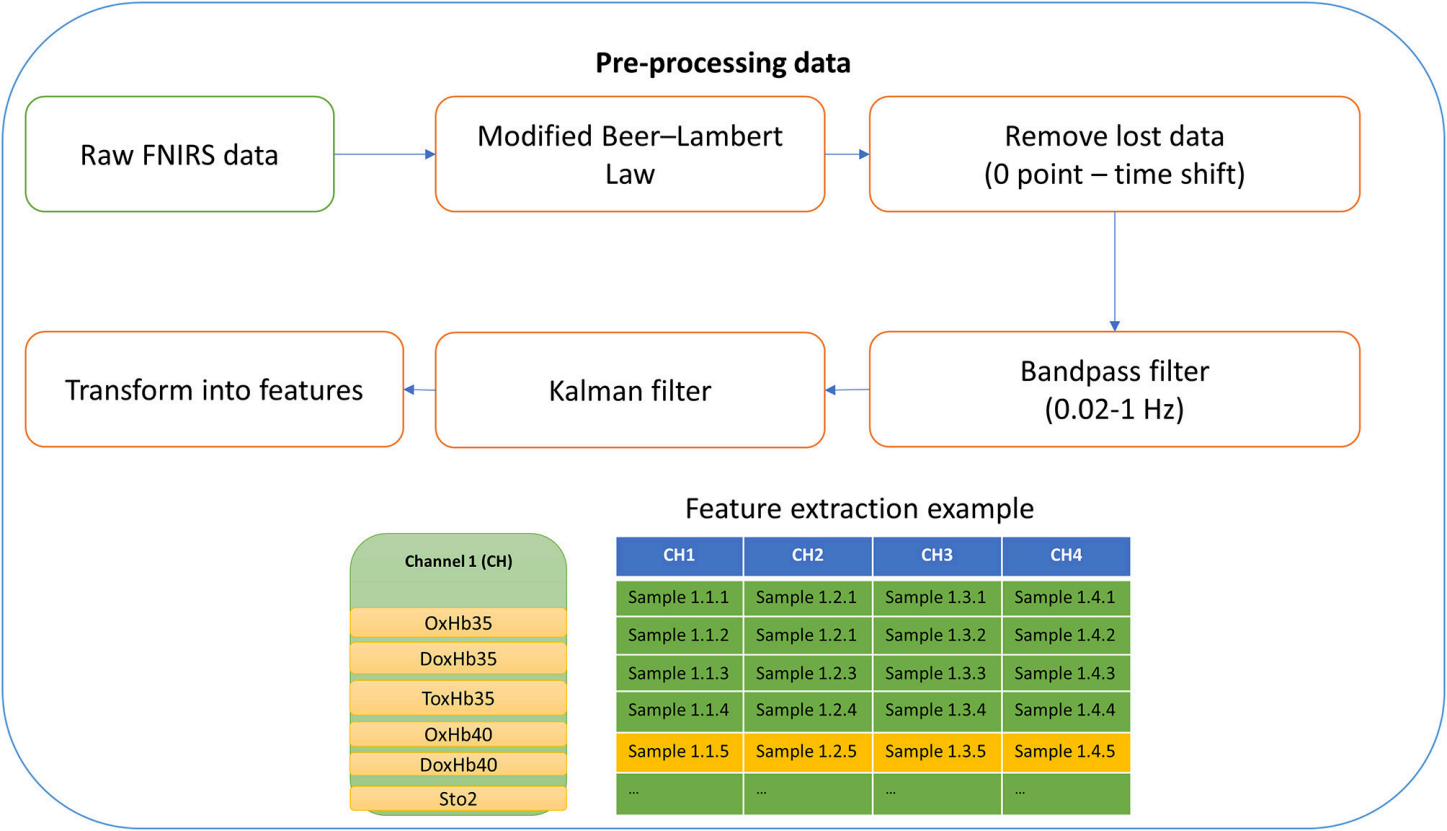
Pre-processing data.

Figure 4 provides an overview of the method we used to preprocess the NIRS data. Because of noise arising from movement artifacts (Cooper et al., 2012; Kirlilna et al., 2013), the raw NIRS data from each channel were preprocessed by using a modified form of the Beer–Lambert Law (Huppert et al., 2016) with removal of lost data (time shift) (Kirlilna et al., 2013), bandpass filtration (0.02–1 Hz) (Ichikawa et al., 2014), and Kalman filtration (Abdelnour and Huppert, 2009), before finally being transformed into features.

**Figure 5:**
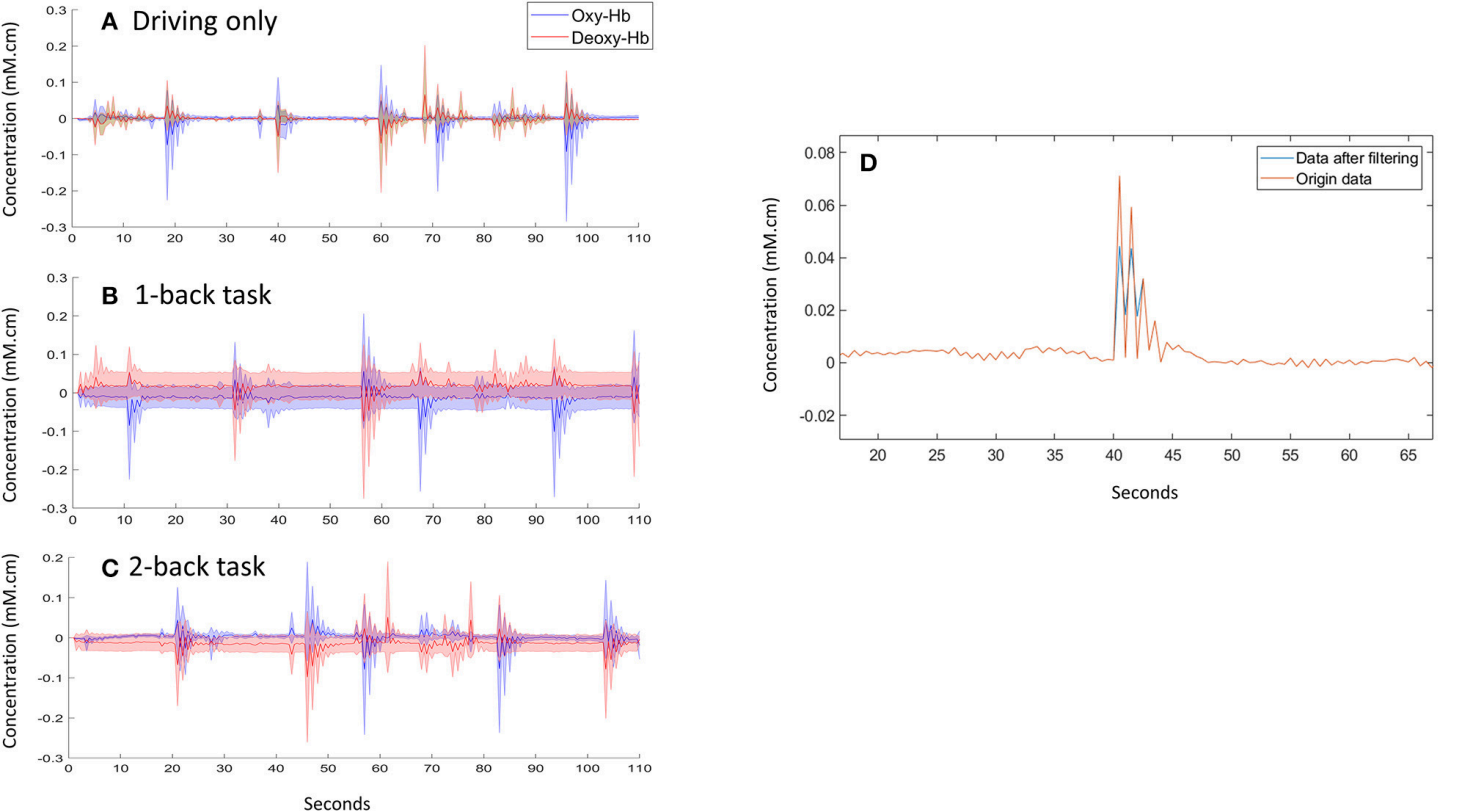
**(A–C)** data collection in one trail for subject 1 **(D)**. Example of filtering data for subject 2.

In our experiment, the raw data of each channel includes (Figure 5): oxyhemoglobin at 35 mm (OxHb35), deoxyhemoglobin at 35 mm (DoxHb35), total oxyhemoglobin (ToxHb35), absolute tissue saturation (StO2), oxyhemoglobin at 40 mm (OxHb40), deoxyhemoglobin at 40 mm (DoxHb40). After filtering, all of the information from NIRS will be transform to 6 features namely OxHb35, DoxHb35, ToxHb35, StO2, OxyHb40, and DoxHb40 for preparing to input for machine learning step.

**Figure 6:**
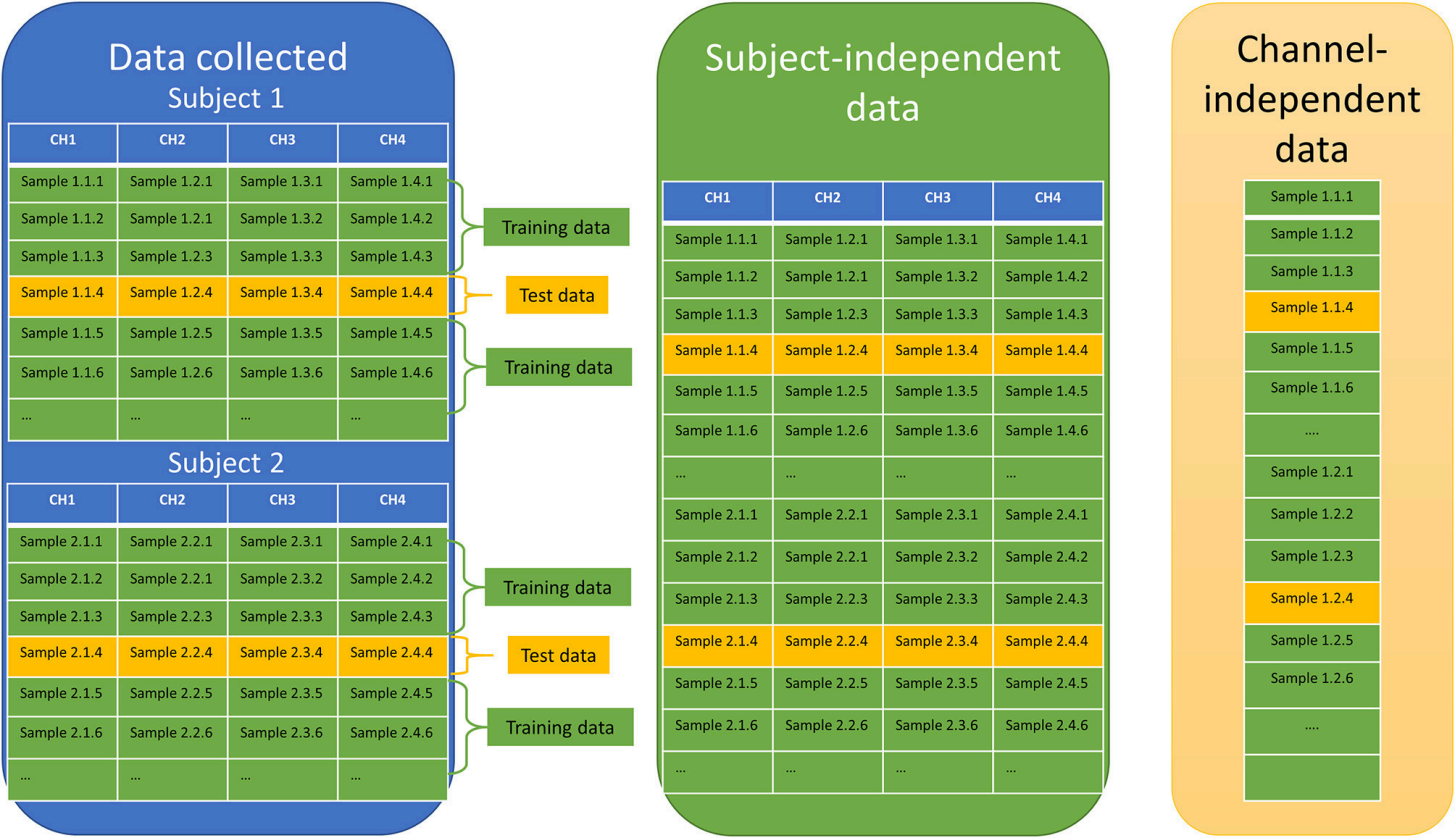
Combining channels.

These features were then processed to create training data and testing data for subject-dependent, subject-independent, channel-independent, and subject-independent plus channel-independent cases. After taking all of the data, they were divided into 75% (training data) and 25% (testing data, which is not used to improve the model, but to measure its predictive performance; Figure 6). Because we used four channels for the forehead, the data combinations were obtained merely by combining the data together. To prevent overfitting during machine learning, a fivefold cross-validation and a principal-component analysis were applied before the data were used to train the system. The fivefold cross-validation was conducted in the following three steps. First, the training data (75% of all data) was split into 5-fold. Second, a model for each fold using all the data outside the fold (75% of the training data) was trained and validated. After that, the features were transformed with PCA to reduce the dimensionality of the predictor space (we applied 5 principal components).

**Figure 7:**
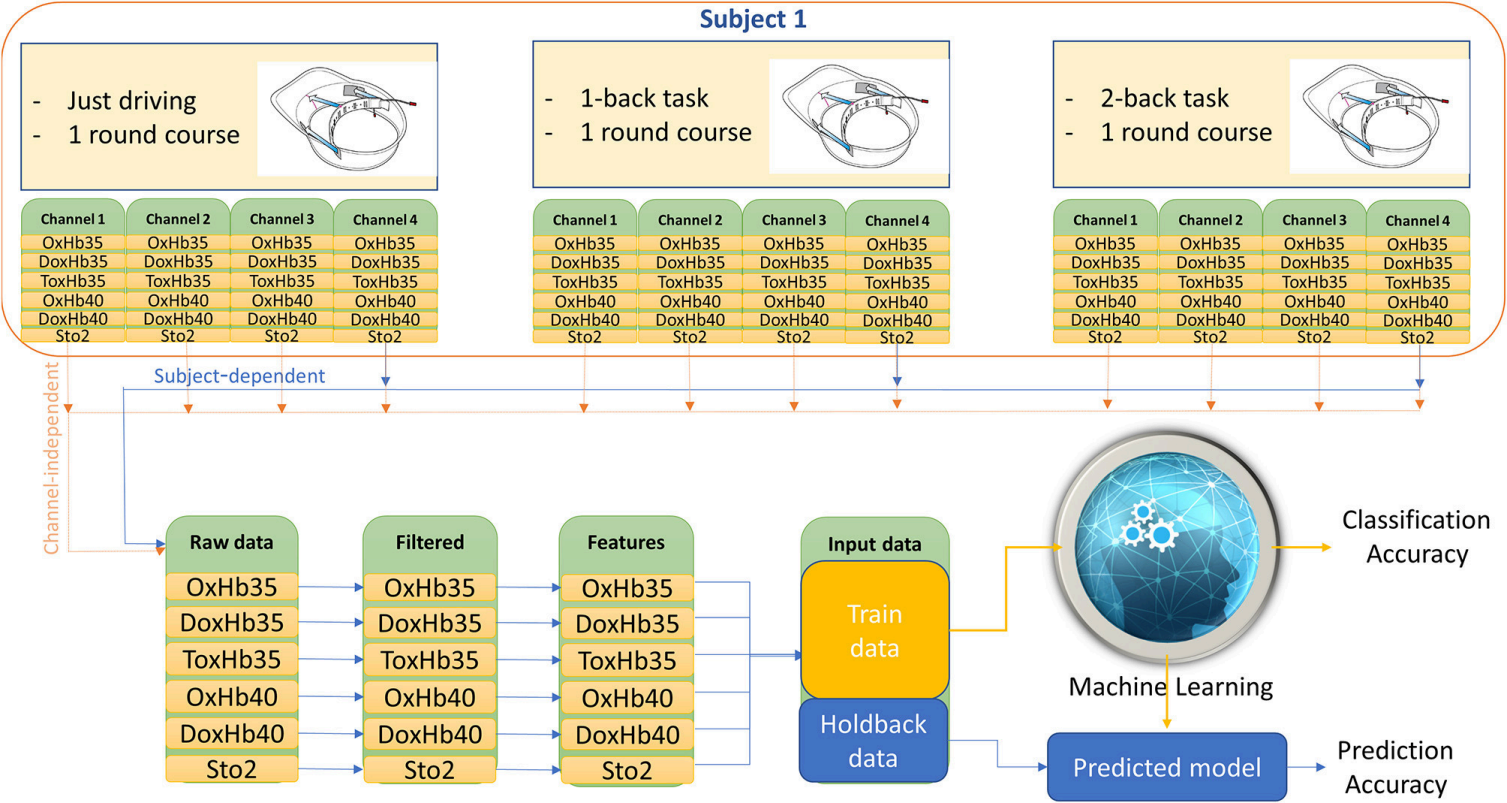
Example for data processing 1.

**Figure 8:**
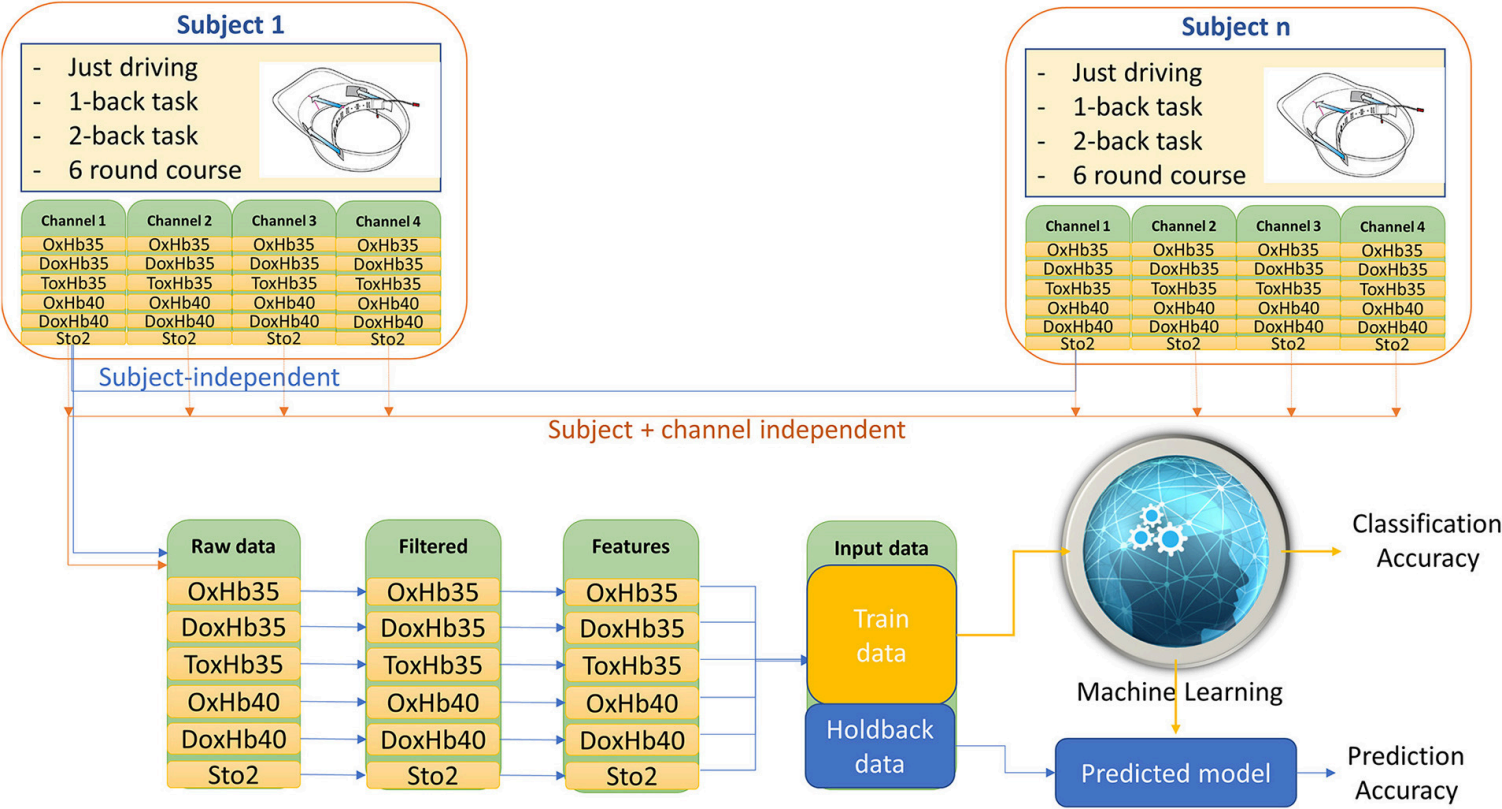
Example for data processing 2.

Definition:

- Subject-dependent (Subject-dependent + channel-dependent; Figure 7): data of each channel for each subject in all task was combined as the input of the machine learning step. Total 20 datasets were prepared for running machine learning.
- Subject-independent (Subject-independent + channel-dependent; Figure 8): data of each channel for all subjects in all task was combined as the input of the machine learning step. Total 4 datasets were prepared for running machine learning.
- Channel-independent (Channel-independent + Subject-dependent; Figure 7): data of all channel for each subject in all task were combined as the input of machine learning step. Total 5 datasets were prepared for running machine learning.
- Subject-independent + Channel-independent (Figure 8): data of all channel for all subject in all task were combined as the input of machine learning step. Only one data set was prepared for running machine learning.

### The classification method

Previous studies on the classification of mental workload from NIRS data have used an SVM (Devos et al., 2009; Ichikawa et al., 2014; Aghajani et al., 2017), linear discriminant analysis (Luu and Chau, 2009), the hidden Markov model (Sitaram et al., 2007; Zimmermann et al., 2013), or artificial neural networks (Chan et al., 2012; Thanh Hai et al., 2013). However, most of these studies involved complicated multichannel NIRS systems. In our study, because of the large number of samples and the low number of channels, we applied supervised learning in MATLAB 2017b (MathWorks Inc., Natick, MA, United States) (Figure 5). We used the 75% of the data to train several well-known models, including the decision-tree model, the discriminant-analysis model, the logistic-regression model, SVMs, nearest-neighbor classifiers, and ensemble classifiers. The performance of these classifiers was determined from the accuracy, as calculated by using the equation shown below:

$$\begin{array}{l} Accuracy\;\left(A_{cc}\right)=\;\frac{\left(TP\;+\;TN\right)}{\left(TP\;+\;FP\;+\;TN\;+\;FN\right)} \end{array}$$

where TP is the number of true positives, TN is the number of true negatives, FP is the number of false positives, and FN is the number of false negatives.

In addition, in case of applying SVMs to perform multiclass classification (just driving vs. 1-back vs. 2-back), the transformation technique was applied to reduce the multiclass classification problem to a set of binary classification subproblems, with one SVM learner for each subproblem. One-vs.-All trains one learner for each class. It learns to distinguish one class from all others will be applied in our case.

The most accurate model for classification was selected and used in the prediction step with the testing data. All parameters of any model were kept the same in both classification and prediction step.

**Figure 9:**
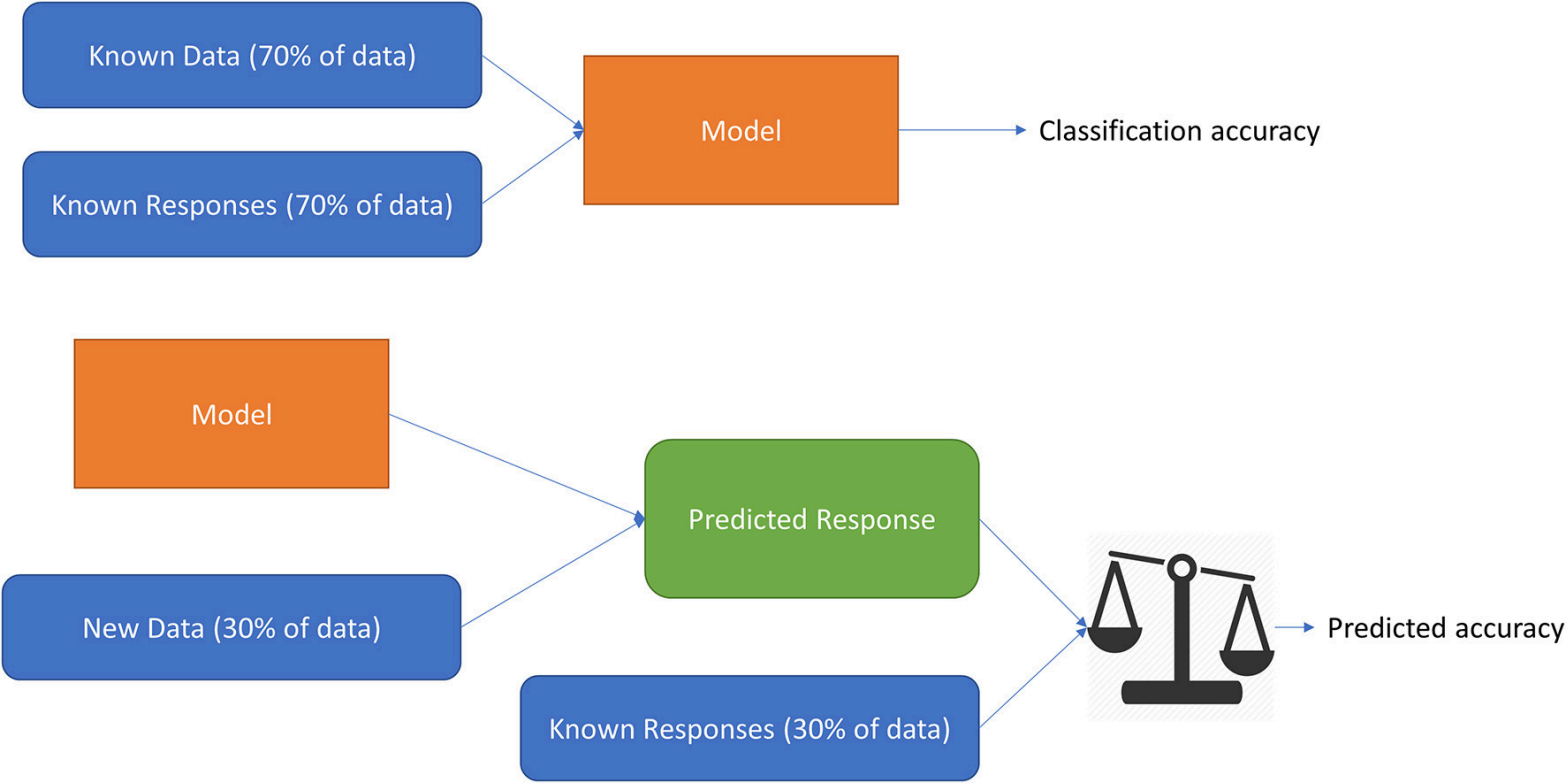
Classification accuracy and prediction accuracy.

Definition (Figure 9):

- Classification accuracy: the accuracy of the classification using 75% of the data for train step. The model of highest accuracy was selected to use in the prediction step.
- Accuracy on the testing data: the accuracy of prediction using 25% of the data for the prediction step. In this step, the prediction model was exported from the selected model in the classification step and apply it for the new data (25% testing data).

## Results

### Subject-dependent classification analysis

Data for each channel for each subject were separated for the purposes of training and prediction. The classification between driving only, driving with a 1-back task, and driving with a 2-back task showed a good performance and a high accuracy (the classification accuracy increased from 81.30 to 95.40%, and the accuracy for the testing data increased from 82.18 to 96.08%) (Video 1). Details of the accuracy are shown in Table 1.

**Table 1:** The classification accuracy (the accuracy on the testing data) (%).

	**Subject 1**	**Subject 2**	**Subject 3**	**Subject 4**	**Subject 5**
Channel 1	94.0 (93.3)	95.4 (96.1)	91.4 (91.3)	92.1 (91.3)	89.7 (91.1)
Channel 2	88.9 (87.9)	92.1 (88.3)	89.7 (89.9)	92.6 (92.3)	87.3 (91.0)
Channel 3	92.9 (90.4)	89.8 (90.6)	91.9 (88.7)	85.2 (82.3)	83.0 (86.4)
Channel 4	94.4 (92.5)	89.9 (90.0)	87.8 (89.5)	85.7 (85.4)	81.3 (82.2)

### Subject-independent classification analysis

**Figure 10:**
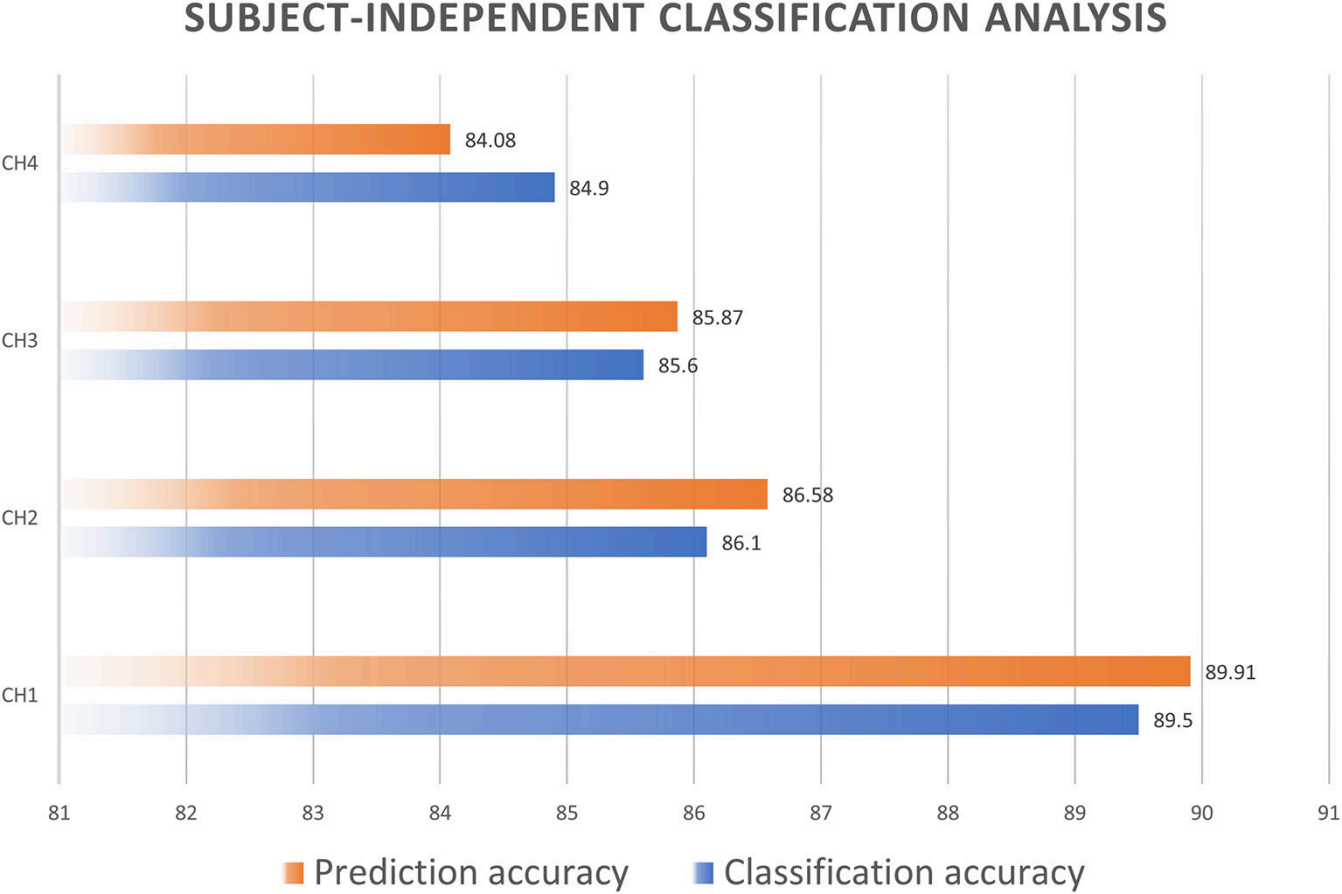
Subject-independent classification accuracy.

With the main arm of comparing the effects of individual characteristics on the accuracy of classification, we also performed a classification with the data for each channel for all subjects. The results are shown in Figure 10. The accuracies in classifying the driver's mental workload from each channel were found to be in the range 84.9 to 89.5%, and the accuracy in predicting testing data increased from 84.08 to 89.91%. These results indicated that individual characteristics affected the accuracy of classification of the mental workload.

### Channel-independent classification analysis

Before combining the data from all channels from the NIRS together, we performed a multiple comparison of oxy-HB and deoxy-HB levels for all the channel data from each subject in the same task by means of a Bonferroni test. The results showed that there was no significant difference between the data from the various channels in the same task (the *p*-value in all cases was >0.05). According to the result, we decided to combine the data from all the channels together when checking the accuracy of classification.

**Figure 11:**
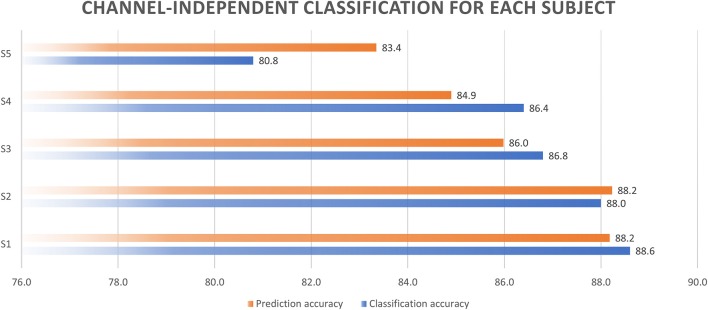
Channel-independent classification accuracy.

First, the data from the four channels for each subject were combined to test the accuracy of classification. The results of this classification are shown in Figure 11. The accuracy of classification increased from 80.8 to 88.6%, and the accuracy on the testing data increased from 83.4 to 88.2%. This shows that acceptably accurate results of classification can be obtained simply by combining the data for the various channels.

### Subject-independent + channel-independent classification analysis

Finally, to examine the potential for creating a system real-time classification of driver cognitive load to prevent accidents, we combined the data from all the channels and all the subjects and we used the combined date to train the classification. We then examined the effect of this combination on the accuracy of classification and on the accuracy of prediction. As expected, the accuracy of classification was 82.9% and the accuracy of prediction was 82.71%, which were similar values to those previously obtained. This showed that that the position of the channel on the forehead did not have a significant effect on the accuracy, and it confirmed that a compact NIRS device can capture the cognitive distraction of a driver, even under naturalistic conditions.

Compare with the result done by Naseer and Hong (2015) and Hong et al. (2015), which was used fNIRS signal, and then show the possibility of the hybrid feature extraction to classification with motor imagery tasks. The highest classification accuracy was around 77.5% with multi-class LDA. Furthermore, the classification of the right—and left—wrist motor imageries also done by Naseer and Hong (2013) using fNIRS information. By reducing the time span within the task period to 2–7 s, the accuracy for classification was increased to 77.56 and 87.28%. Here, we performed the classification with a machine learning algorithm and get better results with accuracy around 82.9% (in the case of subject-independent + channel-independent). The different of the accuracy may depend on the difference in mental workload level, different experiment condition, and so on.

## Discussion

### Model selection for classification of NIRS data

**Figure 12:**
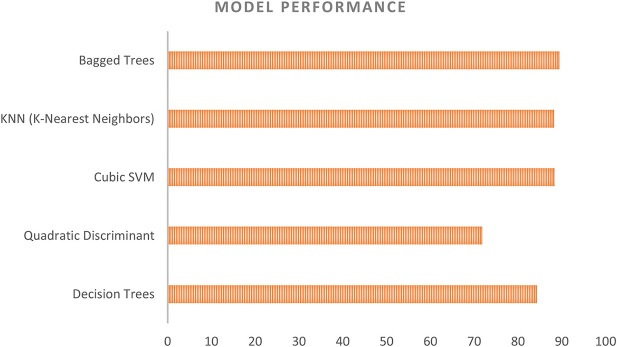
Model performance in Subject-dependent test.

As we have mentioned above, most previous researchers have used an SVM (Devos et al., 2009; Ichikawa et al., 2014; Aghajani et al., 2017), linear discriminant analysis (Luu and Chau, 2009), the hidden Markov model (Sitaram et al., 2007; Zimmermann et al., 2013), or artificial neural networks (Chan et al., 2012; Thanh Hai et al., 2013) to classify mental workload. In this study, we trained the data with some new models, such as the k-nearest neighbors model (k-NN) and the bagged tree (random forests) model, depending on the number of samples. Our results showed that the random forests model provided the highest accuracy, even with large numbers of samples, whereas the cubic SVM showed the worst performance (The average accuracies of each model in all previous analyses are shown in Figure 12). In addition, the k-NN model is also suitable for classifying mental workloads by using NIRS data because of its ability to maintain similar levels of accuracy even when the sample size changes markedly.

The SVM, a well-known method that has been previously applied in various classifications of NIRS data, showed very good performance with small numbers of samples. However, when there were large numbers of samples, the SVM was very slow and its accuracy was low compared with other methods. For example, in the case of a channel-independent test for Subject 1, where the sample size was over 15,000 samples, the accuracy of the SVM was 68.2%, compared with 87.3% for the random forests method and 88.6% for the k-NN classifier. In addition, the SVM took 1,341 s to perform the classification, whereas the k-NN classifier required only 88.6 s. Similar effects were observed in subject-dependent and subject-independent classifications. The sudden reduction in the accuracy of the SVM might arise from differences in the data after data combination, as well as from effects of individual characteristics.

On the other hand, the way to select testing sample also plays a very importance role in the classification. In our case, we selected the testing sample following X → X → X → Y → X → X → X → Y → X …, where X is a sample taken for the 75% and Y is a testing sample. It may make the nearest neighbor classifier that will perform very well, probably because the variation from Y to its neighbors in time (the X before and after Y) will be very low.

We also believe that for higher numbers of subjects, a lower accuracy is attained for subject-independent classification. Consequently, for large numbers of subjects, the machine-learning algorithm should be changed to a deep learning or convolution neural-network algorithm, which can still show good performance with large quantities of data.

### The potential for using NIRS data to evaluate levels of driver mental workload

This study is one example of the application of machine learning in classifying driver mental workload from data obtained with a simple commercial NIRS device, which has a high potential for routine use with drivers because of its acceptable price. However, we believe that the use of a combination of channels for NISR is necessary because signal losses tended to occur often under naturalistic conditions. In some cases, however, one or two channels provided sufficient signals due to the activities of the driver.

The ability to unobtrusively detect changes in mental workload is relevant since high levels of cognitive load can reduce a driver's ability to anticipate and respond to emergent dangers in the driving environment. Broadly considered, these findings suggest various lines of potential research related to the development of advanced driver assistance systems (e.g., a new method to prevent accidents by detecting levels of mental workload that may lead to cognitive distraction), basic human factors insight (exploring the relationship between individual characteristics and objective indicators of mental workload), and mathematical modeling (combining channel, improve accuracy by applying different technical).

In conclusion, as previously suggested (Kopton and Kenning, 2014; Unni et al., 2017), simple NIRS has considerable potential for capturing driver mental workload, especially under naturalistic conditions.

## Limitations

The relatively small sample size used in this study (a total of 5 subjects including one female and four males) could be considered a limitation. While we believe that the NIRS signals were found to be predictive for this small sample under our specific set of conditions, it would be worthwhile to repeat the experiment with a larger sample and a wider range of conditions (e.g., driving track, time of day, gender balance, driver skill level, age, etc.).

## Conclusions

Our study suggested that it is possible to use NIRS data to classify levels of driver mental workload, even in a naturalistic situation. Furthermore, a simple combination of forehead channels was shown to provide acceptably high accuracies of classification. While the fNIRS sensors employed in this study required contact with the participants' skin, the lightweight ball cap configuration was much less intrusive than more traditional electrophysiological measures used in related work. We also confirmed the potential of using machine learning (channel- and subject-independent) to predict possible driver cognitive distraction, a critical factor in road safety.

## Author contributions

AL, HA, FM, and KI generated the idea. AL and HA planned the experiments, collected and analyzed the data, and prepared the manuscript for this study.

### Conflict of interest statement

The authors declare that the research was conducted in the absence of any commercial or financial relationships that could be construed as a potential conflict of interest.

## Supplementary material

Video 1 NIRS toolbox for classifying driver mental workload.
